# Bisphenol A in Chronic Kidney Disease

**DOI:** 10.1155/2013/437857

**Published:** 2013-07-31

**Authors:** Emilio González-Parra, Jose Antonio Herrero, Usama Elewa, Ricardo J. Bosch, Alberto Ortiz Arduán, Jesus Egido

**Affiliations:** ^1^Servicio de Nefrología, IIS-Fundación Jiménez Díaz and IRSIN, Madrid, Spain; ^2^Unidad de Dialisis, Servicio de Nefrología, IIS-Fundación Jiménez Díaz, Avenida Reyes Católicos 2, 28040 Madrid, Spain; ^3^Universidad Autónoma de Madrid, Spain; ^4^Servicio de Nefrología, Hospital Clínico San Carlos, Profesor Martín Lagos s/n, 28040 Madrid, Spain; ^5^Laboratorio de Fisiología Renal y Nefrología Experimental, Departamento de Fisiología, Universidad de Alcalá, Madrid, Spain; ^6^Centro de Investigación Biomédica en Red de Diabetes y Enfermedades Metabólicas Asociadas (CIBERDEM), Spain

## Abstract

Phenols are uremic toxins of intestinal origin formed by bacteria during protein metabolism. Of these molecules, p-cresol is the most studied and has been associated with renal function impairment and vascular damage. Bisphenol A (BPA) is a molecule with structural similarity with phenols found in plastic food and beverage containers as well as in some dialyzers. BPA is considered an environmental toxicant based on animal and cell culture studies. Japanese authorities recently banned BPA use in baby bottles based on observational association studies in newborns. BPA is excreted in urine and uremic patients present higher serum levels, but there is insufficient evidence to set cut-off levels or to link BPA to any harmful effect in CKD. However, the renal elimination and potential exposure during dialysis warrant the monitoring of BPA exposure and the design of observational studies in which the potential health risks of BPA for end-stage renal disease patients are evaluated.

## 1. Uremic Toxins

Multiple molecules accumulate in chronic kidney disease (CKD), are responsible for uremic symptoms, and contribute to increased mortality (uremic toxins). Removal of uremic toxins therefore is accompanied by an improvement in the clinical situation. The term of uremic toxin was created by Piorry in 1847 to indicate the “blood contamination with urine” to refer to the signs and symptoms resulting from kidney disease that increase mortality. Bergstrom [1] proposed that a uremic toxin should be defined as one molecule that meets the following premises:

1. the chemical identity and concentration in biological fluids should be known,
2. the concentration in uremic individuals should be higher than in nonuremic subjects,
3. the concentration should correlate with uremic symptoms, and symptoms should disappear by decreasing the concentration.

Uremic toxins have been classified according to size [2]. Over 350 small uremic toxins have been described with a molecular weight below 500 Da [3]. Medium-sized molecules have a molecular weight between 500 and 5000 Da. Many uremic toxins are bound to proteins which hamper their clearance.

Uremic toxins are responsible for uremic disease. Among the changes that have been directly related to uremic toxins we find progressive loss of renal function, cardiovascular morbidity, and uremic symptoms such as anorexia, vomiting, weakness, sleep disturbances, and neuropathy.

The origin of uremic toxins is multiple. Most uremic toxins originate from the endogenous cellular metabolism. However, there is a growing list of uremic toxins originated in the intestinal microbiota [4]. The diet and the environment may also be sources of uremic toxins [5].

Uremic toxins accumulate progressively as kidney function decreases. There are therapeutic approaches to lower the levels of certain uremic toxins in CKD, such as the use of phosphate binders. Once end-stage renal disease is reached, dialysis contributes to clearance of molecules <50,000 Da. Clearance of small molecules like urea (60 Da) or creatinine (113 Da) depends mainly on diffusion, so they are cleared efficiently by any dialysis membrane or technique. However, medium and large molecules are better cleared by techniques providing high convective transport.

## 2. Protein-Bound Uremic Toxins

A 2008 review found 115 protein-bound uremic toxins [2]. Protein binding impairs dialysis clearance, regardless of its molecular weight [6]. Online hemodiafiltration with reinfusion of ultrafiltrate is the technique that better clears protein-bound toxins [7].

In addition albumin-bound toxins may be taken up by albumin receptors, thus facilitating access into the celland toxicity. Furthermore toxin binding to albumin competes with binding of other molecules typically carried by albumin, interfering with their distribution and metabolism. As an example albumin-bound uremic toxins can increase toxicity of anionic drugs and endogenous molecules such as bilirubin.

## 3. Phenols and Indoles

Phenols and indoles are the best-characterized protein-bound uremic toxins. Both have been related to progression of renal failure and to vascular damage. These toxins are metabolites of protein catabolism by intestinal bacteria, which is greatly increased in patients with CKD. Dietary proteins and peptides are degraded by proteases and peptidases to amino acids. A part of the amino acids reach the colon where they are degraded by intestinal bacteria generating potentially toxic metabolites such as ammonium, amines, thiols, phenols, and indoles. These products of putrefaction in the colon are eliminated in feces although some are absorbed [8].

**Figure 1:**
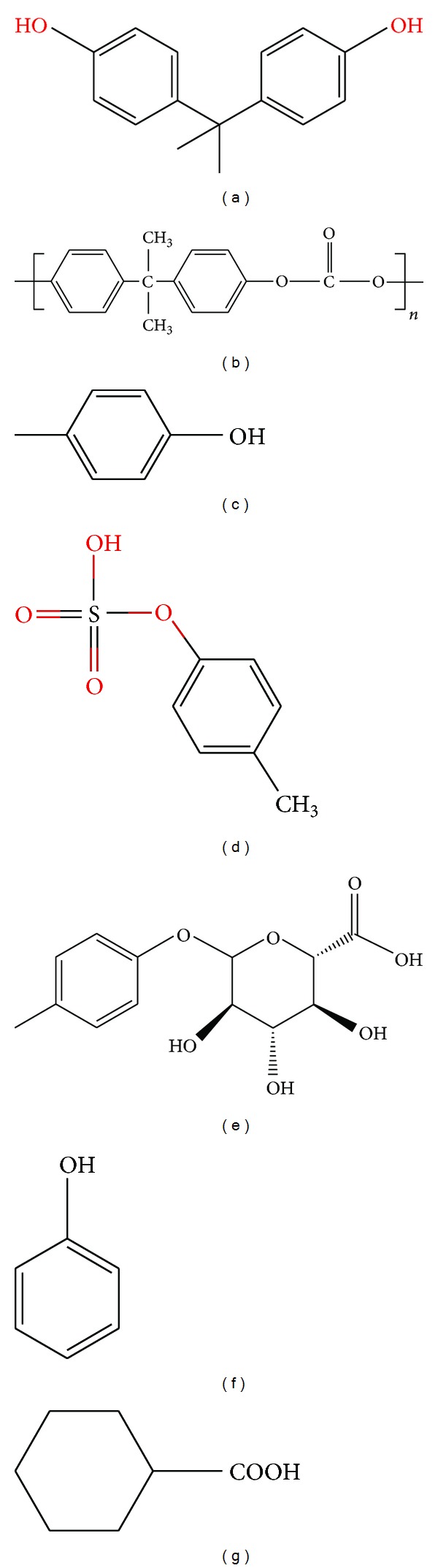
Chemical structures. (a) Bisphenol A (BPA), (b) BPA-containing polycarbonate monomer, (c) p-cresol, (d) p-cresyl sulfate (e) p-cresyl sulfate glucuronide, (f) phenol, and (g) phenylacetic acid.

Among the phenols we must highlight p-cresol and p-cresyl sulfate, phenyl acetic acid, phenol, and polyamines [9] (Figure 1).

- p-Cresol, p-cresyl sulfate, and p-cresol glucuronide: p-cresol is a potent oxidant product of the metabolism of phenylalanine and tyrosine by intestinal anaerobic bacteria. Most p-cresol is conjugated by the gut microbiota to p-cresyl sulfate and in the liver to p-cresylglucuronide. Most p-cresol and p-cresyl sulfate reache the circulation [10]. Free p-cresol (unbound to proteins) has been associated to cardiovascular damage in nondiabetic patients with increased cardiovascular mortality [11]. However, the method used to assess p-cresol levels did not distinguish p-cresol from conjugated p-cresyl sulfate, and there is evidence for the toxicity of p-cresyl sulfate [9].
- Phenol comes mainly from the diet, tobacco, and catabolism of tyrosine and other substrates by intestinal bacteria.
- Phenyl acetic acid results from degradation of phenylalanine and has been associated with impaired immunoregulation [12], oxidative stress [13], and osteoblast dysfunction [14].
- Polyamines are a group of organic cations that include spermine, spermidine, and putrescine, derived from the catabolism of L-arginine and L-ornithine [15]. These three molecules are associated with erythropoietin (EPO) resistance. Polyamine-conjugated proteins accumulate in patients on hemodialysis [16].

Indoles include indoxyl sulfate and indoleacetic acid [9]. Indole is an aromatic heterocyclic structure which is found in many organic compounds such as tryptophan and its metabolites. Intestinal bacteria produce indole and indole acetic acid from tryptophan. Indole is subsequently sulfated to indole sulfate by liver enzymes. These metabolites accumulate in CKD.

- Indoxyl sulphate is a renal and vascular toxin. In hemodialysis patients indoxyl sulfate levels are associated with atherosclerosis [17], endothelial dysfunction, and vascular calcification [18]. Indoxyl sulphate has also been implicated in the progression of renal disease as it may impair cellular antioxidant defenses and be proinflammatory and profibrotic [19].
- Indoleacetic acid has been associated with progression of renal interstitial fibrosis [20].

## 4. Intestinal Uremic Toxins and Uremic Symptoms

Phenols and indoles have been linked to multiple clinical changes in CKD patients. These include the following.

- Progression of chronic renal failure: both indoxyl sulphate as p-cresyl sulfate have been associated with accelerated renal function deterioration [21]. In a prospective study of 268 patients with CKD both molecules were predictive of progression of kidney disease, independently of other cardiovascular risk factors [22].
- Cardiovascular complications: indoxyl sulphate levels were associated with vascular damage and aortic calcification [23]. Indoxyl sulphate is involved in oxidative stress which promotes proliferation of vascular smooth muscle cells [24]. It also increases fibrogenesis and hypertrophy in cardiac fibroblasts [25].
- Anemia: indoxyl sulphate has been linked to renal anemia by interfering with erythropoietin production [26].
- Osteodystrophy: indoxyl sulphate promotes oxidative stress and osteoblast resistance to parathormone (PTH), thus potentially contributing to adynamic bone [27].

CKD patients have an abnormal intestinal microbiota, resulting in disrupted fermentation of carbohydrates and putrefaction of proteins. In hemodialysis patients aerobic bacteria such as enterobacteria and enterococcus are increased and anaerobic bifidobacteria are decreased [28]. Several factors contribute to intestinal dysbacteriosis [8].

- Diet: the dialysis patient eats fewer fibers than the general population, in part due to prescription by healthcare workers to avoid an overload of potassium.
- Constipation: the intestinal transit time increases, partly due to diet and partly to intestinal dysfunction.
- Impaired absorption and metabolism of proteins, carbohydrates, and fats, decreasing the ratio of carbohydrate/protein [29]: among the likely causes are bacterial overgrowth and exocrine pancreatic dysfunction and biliary secretion. Uremia is associated with high plasma levels of pancreatic secretagogue peptides as secretin, gastrin, and an abnormal pancreatic juice composition with decreased bicarbonate and amylase.
- Alteration of intestinal pH with increased intraluminal ammonia.
- Drugs modifying the intestinal microbiota such as antibiotics and phosphate binders.

## 5. Decreasing Intestinal Absorption of Indoles and Phenols in CKD Patients

Evidence available so far suggests that it would be desirable to maintain low levels of phenols and indoles. In CKD patients before renal replacement this involves decreasing their synthesis or interfering with their absorption. Intestinal synthesis may be decreased by use of probiotics or prebiotics, by decreasing colon nitrogen and increasing carbohydrates, and by a diet rich in vegetables and fruits. Probiotics or prebiotics normalize the intestinal microbiota increasing saccharolytic and decreasing proteolytic bacteria. Both products reduce levels of phenols in both healthy individuals and in patients on hemodialysis [30, 31]. Acarbose reduces the generation of phenols in colon by increasing carbohydrate availability [32]. A diet rich in vegetables and fruits also reduces the synthesis of phenols [33], but this diet is not usually used in CKD patients for the high risk of hyperkalemia. AST-120, an oral absorbent microsphere, decreased the absorption of p-cresol [34] and delayed CKD progression [35].

## 6. Bisphenol A (BPA)

Bisphenol A (BPA) is an environmental toxin containing phenolic rings with structural similarity with phenols (Figure 1). While the origin of the toxins differs, the metabolism and side effects of BPA may have common characteristics with phenols of intestinal origin. BPA is eliminated by the kidney, and increased blood levels have been observed in CKD.

BPA was synthesized in the thirties as a synthetic estrogen [36, 37] but was displaced by diethylstilbestrol. Currently BPA is used as an additive in plastics and resins. BPA adds hardness, clarity, lightweight, and resistance to temperature to polycarbonate plastic and epoxy resins. Polycarbonates are used in plastic containers commonly used in the food industry and at home, such as plastic bottles, lenses, and medical devices. BPA epoxy resins are used as coating in food and beverage cans. However, due to the potential impact on health, in Japan epoxy coating was replaced by a polyester film [38]. BPA is also used in polysulfones and polyether ketones, as an antioxidant in some plasticizers and as a polymerization inhibitor in polyvinyl chloride (PVC).

BPA is a chemical switch in endocrine processes, and may impact reproduction, weight, and development. BPA acts like a hormone, altering cellular function at very low concentrations, with the maximum safe levels of 5 mg/kg/day [39].

**Figure 2:**
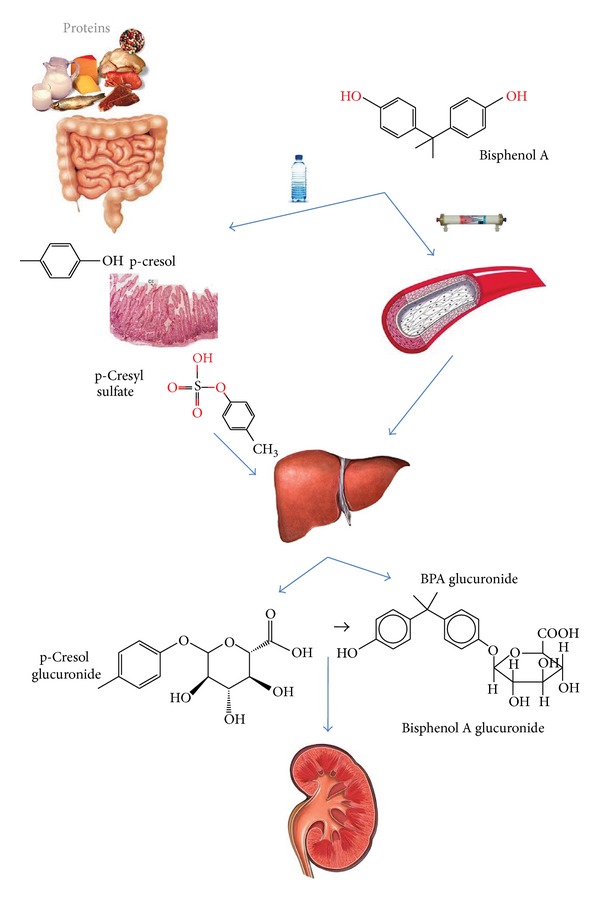
Origin and metabolism of microbial (p-cresol) and environmental (BPA) phenols. Both can be absorbed in the digestive tract, conjugated in the liver, and eliminated by the kidneys. BPA may also enter the blood from dialysis membranes.

BPA may be absorbed in the gastrointestinal tract after ingesting products packed in plastic containers. Like intestinal phenols, BPA is conjugated by glucuronic acid in bowel and liver and excreted in urine as BPA-glucuronide [40] (Figure 2). Exposure may occur by a nonoral route, so it is considered a toxic environmental origin. Inhalation of BPA at concentrations > 5.1 mg/m^3^ (0.11 to 0.54 ppm) can cause coughing and bronchospasm that may persist for several days. BPA can also cause conjunctivitis, periorbital edema, skin redness, and roughness.

The EU banned baby bottles with BPA in June 2011 amid concerns that BPA may impact neurological and reproductive development. Studies in animal and cell models have found these alterations [41–45]. Human data are abundant, but less consistent, reporting observational associations that cannot prove causality [46–50]. Certain groups, such as pediatricians, have expressed interest in a primary prevention [46]. However, neither the US Food and Drug Administration (FDA) nor the EU has found sufficient evidence to ban the widespread use of BPA in the food and beverage industry [51]. The FDA has concluded that BPA is safe but in the absence of conclusive scientific criteria has recommended reducing exposure as much as possible [52]. BPA has not been banned on the basis of the following criteria: high cost of banning its use, high BPA doses used in animal studies, lack of human interventional studies, different metabolism in humans and animals, and complete renal elimination immediately after intake. However, the last criterion would not apply to patients with CKD, and the possibility that BPA may be an environmental uremic toxin requires further study.

## 7. BPA Toxicity in Experimental Animals

BPA can disrupt the development of newborn animals and the immune system and may be carcinogenic. Evidence from animal studies also shows alterations in adult animals exposed to BPA resulting from estrogenic activity [41] or causing liver damage [42], pancreatic damage [43], thyroid disorders [44], or obesity [45]. 

There is direct evidence of neurological and endocrine toxicity of BPA in rodents and zebra fish [53]. BPA can influence the development of the embryonic zebra fish brain including the hypothalamus, telencephalon, and preoptic areas [54]. Exposure in utero or perinatally to BPA can lead to permanent behavioral disorders in rodents, including increased levels of aggression and anxiety and alterations in learning, memory, exploration, and emotional responsiveness [55]. Several mouse studies concluded that maternal exposure to low-dose BPA has long-term consequences on neurobehavioral development [56] and neonatal exposure to BPA can affect brain morphology and neuronal adult phenotypes [57].

Exposure to BPA has persistent effects on body weight and adiposity [58]. In rats, perinatal exposure to drinking water containing 1 mg/L BPA increased adipogenesis in females at weaning [59].

The effects of neonatal BPA exposure on reproductive organs in mice depended on the level of BPA, 2 mg/kg increased the adult prostate weight [60] and 10 mg/kg disrupted prostate development [61]. Exposure of rats to 10 mg/kg BPA increased susceptibility to prostate carcinogenesis in adults [62]. Male rats exposed to BPA have lower sperm counts and testosterone levels with a significant effect on fertility [53, 63].

## 8. BPA Toxicity in Humans

There are no studies linking serum BPA to disease in humans, so it is unknown whether there are cutoff levels that may be worrisome. Available studies have evaluated BPA exposure by assessing urinary BPA [52]. The evidence is observational and no firm conclusions can be reached as to causality.

Exposure estimation based on urinary excretion may not be valid in CKD [52]. In CKD it is unclear whether low urinary excretion reflects low exposure or increased retention. Exposure to BPA containers, such as bottles, for 7 days increases urinary BPA excretion in normal subjects from 1.2 mcg/g creatinine to 2 mcg/g [64].

In newborn infants greater exposure to BPA was associated to altered behavior [47]. Prenatal exposure to BPA, evaluated by maternal urinary BPA excretion (1–4 mcg/L), may be associated with behavioral disorders at 2 and 3 years of age [65]. These values represent 1–8 mcg for an average adult of 70 kg. Comparatively, deleterious effects in mice required from 60 to 300 mcg BPA for a 20 g animal. Thus, the huge dose used for animal experiments may not be relevant to the human situation.

Several studies have linked BPA exposure to the development of obesity, insulin resistance, metabolic syndrome, diabetes, and atherosclerosis [48–50]. An epidemiological association was observed between high urinary BPA and cardiovascular disease. Each increase in urinary BPA of 4.5 mcg/L was associated with a 13% increase in the incidence of coronary heart disease in 10 years, although the significance was lost after adjustment for traditional cardiovascular risk factors [66]. The loss of the association when adjusted for cardiovascular risk factors suggests that higher urinary BPA may be related to high-risk habits and speaks against a causal association between BPA and cardiovascular risk. In healthy adult Americans' urinary BPA levels >4 mcg/L were associated with a 50% increase in the prevalence of hypertension compared with levels <1.5 mcg/L [67]. Urinary BPA levels >1.4 mcg/L were associated with a 23% higher risk of microalbuminuria than in healthy adults with levels <0.5 mcg/L [68, 69].

## 9. Accumulation of BPA in CKD

Published evidence on BPA in dialysis or CKD patients is scarce and limited to the observation of increased blood BPA levels. However, there is no evidence linking these levels to any particular adverse effect or outcome, no cutoff values have been defined, and no studies have shown that decreasing the BPA levels has any impact on outcomes. The National Health and Nutrition Examination Survey 2003-6 (NHANES III) observed in 2573 patients a decrease of urinary excretion of BPA with renal function impairment. However, this was only significant in women and only when the MDRD formula was used to estimate GFR: it was not observed with CKD-EPI formula [70]. The meaning of these data is uncertain: low urinary BPA excretion may reflect low exposure to BPA (which would be desirable) or retention of BPA by kidney disease (which would not be desirable). In this sense, the lower levels of urinary BPA were associated with lower risk of microalbuminuria [68] or hypertension [60], suggesting that it may reflect less exposure to BPA. By contrast increased serum BPA with decreasing renal function and higher levels in hemodialysis was observed in a smaller study of 32 CKD patients [71], suggesting that BPA may accumulate in CKD. One of the arguments by government agencies for considering the safe use of BPA in the general population is the almost complete urinary elimination of the conjugated molecule [72]. The rapid urinary excretion decreases the risks of exposure to BPA. For this reason, studies on the potential consequences of BPA accumulation in CKD patients are needed.

BPA is found in the shell (polycarbonate) and membranes of some commonly used dialyzers. The amount of BPA in polyester polymer alloy (PEPA) is 12.2 mcg/g, in polysulfone 8 to 34.5 mcg/g, in polycarbonate 47.2 mcg/g, in polymethylmethacrylate 0.008 mcg/g, in cellulose triacetate 0.008 mcg/g, and in cellulose 0.016 mcg/g [71, 73]. 

BPA may be released from dialyzers, although values for BPA release are lower than stipulated by health authorities. Under experimental conditions release of BPA is higher from dialysis filters perfused with blood (0.2 to 0.7 ng/mL) than when perfused with saline (0.1 to 0.2 ng/mL) [74]. This difference has been attributed to the effect of blood hydrophobic components such as lipids or lipoproteins. The brand and sterilization method may also impact BPA release. The highest BPA levels were released from steam-sterilized polysulfone dialyzers with a polycarbonate shell (0.2 to 0.7 ng/mL) [75]. The release of BPA is lower when polysulfone membranes are perfused with water (3.8–142 ng/module) than when they are perfused with bovine serum (141–2090 ng/module) [73].

What are the clinical consequences of these experimental observations? Serum BPA is virtually undetectable when renal function is normal. Mean BPA values in patients with CKD were reported to be 0.23 ng/mL. Higher BPA values were reported in patients dialyzed with first generation polysulfone membranes (4.83 ± 1.94 ng/mL) and increased postdialysis (6.62 ± 3.09 ng/mL), while lower values were found in patients using second generation polysulfones [71]. In another cross-sectional study of 152 prevalent patients with CKD, BPA plasma levels started to rise in patients with CKD stage 3 and were >6-fold higher in hemodialysis patients than in patients with CKD stage 5 not yet on dialys (10.0 ± 6.6 versus 1.6 ± 1.8 ng/mL; *P* < 0.001). The very high levels in hemodialysis patients may be related to inefficient removal due to the high protein-bound fraction of plasma BPA (74 ± 5%) [76]. In a randomized controlled study of 18 hemodialysis patients subjected successively to 4-week dialysis with low-flux polysulfone, high-flux polysulfone, and high-flux polyethersulfone, the plasma BPA concentrations were very high in hemodialysis patients (range 9.1 ± 4.5–12.0 ± 6.0 ng/mL versus ≤0.2 ± 0.1 ng/mL in normal renal function controls; *P* < 0.001) but did not change during hemodialysis with any of the three dialyzers in the course of a single treatment or over a period of 4 weeks [76].

A single study did not observe changes in BPA levels during peritoneal dialysis in 4 patients [77].

## 10. Conclusions

BPA is an estrogenic endocrine disruptor molecule of phenolic structure used in plastics, which has renal elimination, and builds up when the glomerular filtration rate decreases. Hemodialysis patients may be exposed to BPA from the environment and also from hemodialysis filters. However, to date no adverse effects have been linked to BPA in dialysis patients. Furthermore it is unknown whether the removal of BPA from these patients is associated with an improved outcome. Studies should be designed to answer these questions before BPA can be added to the list of bona fide uremic toxins [78]. This requires validated and standardized methods for measuring serum BPA as well as careful prospective studies assessing predefined endpoints.
